# An eMERGE Clinical Center at Partners Personalized Medicine

**DOI:** 10.3390/jpm6010005

**Published:** 2016-01-20

**Authors:** Jordan W. Smoller, Elizabeth W. Karlson, Robert C. Green, Sekar Kathiresan, Daniel G. MacArthur, Michael E. Talkowski, Shawn N. Murphy, Scott T. Weiss

**Affiliations:** 1 Massachusetts General Hospital, Boston, MA 02114, USA; skathiresan1@mgh.harvard.edu (S.K.); macarthur@atgu.mgh.harvard.edu (D.G.M.); mtalkowski@mgh.harvard.edu (M.E.T.); 2 Partners Personalized Medicine, 65 Landsdowne Street, Cambridge, MA 02139, USA; ekarlson@partners.org (E.W.K.); rcgreen@genetics.med.harvard.edu (R.C.G.); snmurphy@partners.org (S.N.M.); restw@channing.harvard.edu (S.T.W.); 3 Broad Institute of MIT and Harvard, 415 Main Street, Cambridge, MA 02142, USA; 4 Brigham and Women’s Hospital, 75 Francis Street, Boston, MA 02115, USA

**Keywords:** Partners Personalized Medicine, personalized medicine, eMERGE, Partners Biobank, biorepository, precision medicine, electronic medical records, genomics

## Abstract

The integration of electronic medical records (EMRs) and genomic research has become a major component of efforts to advance personalized and precision medicine. The Electronic Medical Records and Genomics (eMERGE) network, initiated in 2007, is an NIH-funded consortium devoted to genomic discovery and implementation research by leveraging biorepositories linked to EMRs. In its most recent phase, eMERGE III, the network is focused on facilitating implementation of genomic medicine by detecting and disclosing rare pathogenic variants in clinically relevant genes. Partners Personalized Medicine (PPM) is a center dedicated to translating personalized medicine into clinical practice within Partners HealthCare. One component of the PPM is the Partners Healthcare Biobank, a biorepository comprising broadly consented DNA samples linked to the Partners longitudinal EMR. In 2015, PPM joined the eMERGE Phase III network. Here we describe the elements of the eMERGE clinical center at PPM, including plans for genomic discovery using EMR phenotypes, evaluation of rare variant penetrance and pleiotropy, and a novel randomized trial of the impact of returning genetic results to patients and clinicians.

## 1. Introduction

As enthusiasm for the prospects of personalized or precision medicine has grown, there has been increasing interest in capitalizing on two parallel developments. First, advances in genomic medicine and pharmacogenomic research are providing an expanding catalogue of genetic predictors of disease and health outcomes. At the same time, the increasing integration of electronic medical records (EMRs) into medical practice has created a growing repository of longitudinal data capturing real-world clinical phenotypes. In combination, these parallel developments provide a unique engine for discovery and implementation efforts in genomic medicine [1]. The Electronic Medical Records and Genomics (eMERGE) network was initiated in 2007 by the National Human Genome Research Institute (NHGRI) to facilitate the research integration of EMRs and genomics [2]. In its initial phase (eMERGE I), the network consisted of five sites with biorepositories linked to EMRs, expanding to nine sites by 2012 (eMERGE II). The network has played a major role in developing best practices for EMR-based genomic research, advancing methods for extracting and validating phenotypic data using semi-automated algorithms, and demonstrating the value of phenome-wide genetic association analyses (PheWas) [3].

In 2014, NHGRI issued a request for applications (RFA) for eMERGE Phase III with a primary goal of facilitating implementation of genomic medicine by defining health outcomes associated with rare variants in clinically relevant genes. The eMERGE III effort aims to enhance research in several domains: the discovery of pathogenic rare variants, characterizing their phenotypic effects using validated EMR data, reporting actionable variants to patients and clinicians to improve clinical care and clinical outcomes, and assessing the medical and ethical/legal/social implications of returning these results. Investigators at Partners HealthCare responded to this RFA by leveraging the investment and expertise in informatic, genomic and personalized medicine research ongoing at Partners Personalized Medicine (described below). In 2015, the Partners site was funded to join the eMERGE III Network, and the following sections provide an overview of the establishment and aims of the Partners eMERGE Clinical Center.

## 2. Infrastructure for a Clinical eMERGE Site at Partners HealthCare

### 2.1. Partners HealthCare Personalized Medicine

Partners HealthCare System (PHS) is a not-for-profit, integrated health care system in Boston, Massachusetts founded by Brigham and Women’s Hospital (BWH) and Massachusetts General Hospital (MGH) and includes community and specialty hospitals, a physician network, community health centers, home care and other health related services. PHS is the largest health system in Massachusetts (MA) and cares for approximately 4 million patients. The composition of the covered lives in terms of race, gender and age is reflective of eastern MA. Partners Personalized Medicine is a center dedicated to translating personalized medicine into clinical practice at Partners HealthCare. The components of the center are: The Laboratory for Molecular Medicine (LMM) (see Aronson *et al.* [4]), The Translational Genomics Core (sequencing and genotyping), the Partners Biobank and the Research Patient Data Registry all of which are linked by a common IT infrastructure.

### 2.2. EMR Infrastructure: The Research Patient Data Registry

At PHS, research utilizing EMRs is implemented through the Research Patient Data Registry (RPDR). The RPDR is a data warehouse that gathers data from multiple hospital electronic record systems at Partners HealthCare and stores it in a SQL Server database. Researchers may query the RPDR using an online query tool for aggregate totals and, with proper IRB approval, may obtain detailed medical record information. Security and distribution of patient data is controlled and audited by the RPDR according to IRB and HIPAA guidelines; all patient identifiers are encrypted throughout the database. RPDR currently contains data on 4 million patients, with 75 million encounters, and approximately 900 million distinct, coded clinical facts stored in the database dating back to 1992 including demographic data, diagnoses (e.g., ICD-9 codes), procedures (e.g., CPT codes), pharmacy data (e.g., RxNorm), inpatient and outpatient encounter information, provider information, laboratory data, imaging and pathology data.

### 2.3. Partners HealthCare Biobank

As described elsewhere in this issue (Karlson *et al.* [5]), the Partners Biobank is a collection of DNA, serum, and plasma samples fully consented for: (a) broad use for genomics, biomarker, epidemiology research; (b) linkage with RPDR and survey data; (c) re-contact for phenotyping; and (d) data sharing with dbGAP and approved collaborators. Of importance, given the focus of eMERGE III, the Biobank consent form provides for the return of medically actionable genetic results. At this writing, the Biobank has enrolled more than 32,000 participants, on the way to a target of 75,000 by 2018. To facilitate genomic research and participation in eMERGE, the Biobank undertook a plan to conduct genomewide genotyping of 25,000 samples. For this purpose, the Biobank selected a new Illumina Multiethnic Beadchip array comprising 1.8 million SNPs with common and rare variation including and a custom set of more than 40,000 loss-of-function (LoF) variants.

### 2.4. Tools for EMR-Based Phenotypic Definition and Validation

Investigators at the Partners eMERGE center have extensive experience with the definition and validation of EMR-based phenotypes and their application to population-based and genomic research. This has included the application of natural language processing of EMR data using the Informatics for Integrating Biology and the Bedside (i2b2) toolkit [6]. Using *i2b2* methods (Table 1), we have developed and validated EMR algorithms with high positive predictive value (PPV) for the diagnosis of: (a) coronary heart disease (CHD), congestive heart failure (CHF), hypercholesterolemia; (b) neuropsychiatric diseases (BD, major depressive disorder, MDD); and (c) immune diseases (RA, inflammatory bowel disease (IBD), and multiple sclerosis (MS) (e.g., see [7,8]). For example, we developed an algorithm designed to accurately identify all patients with RA*.* From more than 4 million patients in the RPDR, we built an RA dataset of subjects with *any* ICD9 diagnostic code for RA or positive anti-CCP antibody (highly specific for RA), developed an algorithm using codified data and narrative data extracted with natural language processing (NLP) to classify subjects with a high probability of RA, and applied the algorithm to the RA Mart to validate positive predictive value (PPV) (the proportion of subjects classified as cases by the algorithm who are true cases). Using a logistic regression approach with the adaptive LASSO penalty, the final algorithm yielded an area on the receiver operating curve (AUC) of 0.94, with a sensitivity of 63% and a PPV of 94% if specificity was set at 97%. PPV for the algorithm in an independent validation set of patients classified as having RA was 94%. The *i2b2* method demonstrated a dramatic improvement in PPV compared with the use of ≥3 ICD-9 codes (54% *vs.* 94%). We demonstrated portability of the algorithm at two eMERGE sites: AUC was 92% for Northwestern University and 95% for Vanderbilt. As another example, we used NLP and coded data to develop algorithms for BD and controls using the *i2b2* method. These algorithms were then validated against direct diagnostic interviews conducted by trained clinicians blinded to case and control status. With a specificity of 95%, the algorithm achieved a PPV of 0.85 and the PPV for controls was 1.0.

**Table 1:** i2b2 method for defining disease phenotype algorithms.

Steps	Task	Team Member
1	Randomly select 400 subjects with ICD-9 code	Programmer
2	Review charts, confirm diagnosis for Training Set	Domain expert
3	Create custom list of concepts relevant to disease	Domain expert
4	Extract EMR data to create codified variables	Programmer
5	Create custom list of NLP variables	Domain expert
6	Map variables UMLS concept unique identifier (CUI)	Informatician
7	Extract CUIs from narrative text in EMR using NLP	Informatician
8	Run LASSO regression with codified + NLP variables predicting disease status in Training Set	Statistician
9	Set specificity at 97%, select predicted probability among Training Set to achieve >90% PPV	Statistician
10	Apply algorithm to remaining Biobank subjects (excludingTraining Set)	Statistician
11	Randomly select 100 subjects for Validation Set	Programmer
12	Perform chart review in Test Set, define PPV	Domain expert

To enable genomic research at the Partners Biobank, we created a Phenotype Discovery Center (PDC). that integrates data from RPDR, health information surveys, and genotype results into a Biobank Portal. The Biobank Portal combines specimen data with EMR data in a SQL Server database with a web-based application that allows users to perform queries, visualize longitudinal data with timestamps, perform PheWAS based on >1500 clinically grouped ICD9-CM codes, query phenotypes defined by *i2b2* algorithms, perform automated NLP, and request samples from cases and matched controls. Data in the Biobank Portal database includes narrative data from clinic notes, text reports (cardiology, pathology, radiology, operative, discharge summaries), codified data (e.g., demographics, diagnoses, procedures, labs and medications) as well as patient-reported data from the health information survey on exposures and family history. The PDC has developed new NLP tools based on terms extracted from Wikipedia, and Medscape to create automated custom dictionaries of UMLS CUI codes for 8 phenotypes. Algorithm development steps: (1) filtered Biobank subjects by presence of the ICD9-CM billing code; (2) randomly selected 100 subjects with each code; (3) chart reviews to define disease status in a Training Set; (4) automated feature extraction and feature selection to EMR narrative text; (5) LASSO penalized regression with CUI features predicting disease status in Training Set; (6) applied the algorithm to remaining subjects to define disease phenotype set. Performance characteristics of the automated feature extraction algorithms demonstrated similar performance to *i2b2* algorithms (at PPVs = 90, AUCs 0.91–0.99). Validated phenotypes are available in the Biobank Portal user interface for genotyped Biobank participants.

## 3. Scientific Aims of the eMERGE Clinical Center at Partners Personalized Medicine

In response to the eMERGE III RFA, we designed three scientific aims in line with the goals of integrating genomics and EMR data to evaluate the phenotypic impact of rare variants as well as the implications for health-related outcomes of returning clinically-relevant genetic results.

### 3.1. Discovery: Detecting Association of Common and Rare Variants with EMR-Based Phenotypes

The availability of genomewide data for biorepository participants was a prerequisite for participation in the eMERGE III Network. As noted earlier, the Partners Biobank has undertaken array-based genotyping of common and rare variants for 25,000 participants. The genotyping array selected for this work is a custom version of the Illumina Multiethnic Beadchip (MEGArray) that includes 1.8 million SNPs with exome content and supplemented with a custom panel of more than 40,000 LoF variants derived from the sequencing of more than 90,000 exomes through the Exome Aggregation Consortium (ExAC) (http://exac.broadinstitute.org/about). Genotyping began in March 2015 and is expected to be completed for 25,000 participants by March 2016. The largest share of genotyping is being performed at the PPM Translational Genomics Core facility.

Genetic association analyses will focus on discovering clinically actionable variation in three domains of disease for which the Partners eMERGE team has extensive experience in clinical and genomic studies: cardiovascular, neuropsychiatric, and immune-mediated disease. Common variant analyses will focus on previously identified associations to minimize multiple comparisons. However, GWAS will be performed for the more than 40 core phenotypes developed across the eMERGE network. Loci first identified as risk loci for rare variants have sometimes been found to also harbor common variants (and *vice versa*); thus being able to combine evidence from these variant types may increase power. Power analyses indicate that we will have 80% power to detect rare variants with allelic odds ratios of at least 1.29 for most of the primary phenotypes of interest.

### 3.2. Evaluating Penetrance and Pleiotropy of Rare Variants

A major focus of eMERGE III is to evaluate the clinical relevance of rare variants using EMR phenotypes. This effort will involve sequencing of approximately 100 genes across the Network. These will likely include genes identified by the American College of Medical Genetics (ACMG) as warranting return of secondary findings in clinical sequencing [9], the Pharmacogenetics Research Network’s (PGRN) list of actionable pharmacogenetic genes, high impact common GWAS variants, and other eMERGE site nominations. The aim of our research will be to characterize the penetrance and pleiotropic effects of these genes using EMR-data across the eMERGE Network. The eMERGE Network will aggregate samples for sequencing, and the Partners site will contribute at least 2500 DNA samples to this effort. We will assess of the penetrance of pathogenic variants (PVs) in selected genes for diseases known to be associated with those variants by determining the prevalence of the disease among individuals carrying the PV. In addition, it is increasingly clear that pleiotropy (the association of individual genetic variants with multiple phenotypes) is a common and important phenomenon [10]. The broad range of phenotypes captured in EMRs has enabled phenome-wide association studies (PheWas) that provide a powerful opportunity to assess pleiotropic effects. Previous research from eMERGE investigators has defined “PheWas codes” that increase power by grouping clinically-linked diseases [11]. We will use PheWAS codes to identify variants with pleiotropic effects. In addition we will define pleiotropy as association with multiple phenotypes within each group of cardiovascular, neuropsychiatric and immune disease defined by our Phenotype Discovery Center automated algorithms.

The Partners team proposed to focus on genes of clinical relevance to the three disease areas (cardiovascular, neuropsychiatric, and immune-mediated) emphasized by our Center. Examples of relevant genes in these domains are summarized briefly below.

#### 3.2.1. Cardiovascular Genes

Familial hypercholesterolemia (FH), one of the most common monogenic disorders, accounts for an estimated 20% of myocardial infarctions under age 45 and as many as 5% under age 60 despite the availability of effective treatment. FH results in elevated low-density lipoprotein cholesterol (LDL-C) levels and a greater than 20-fold increased risk for CHD. The principal causes of FH are mutations in *LDLR*, followed by *APOB* and *PCSK9*. Work by our group and others suggest that approximately 1 in 115 individuals carry a pathogenic mutation in *LDLR*. Using sequencing, we evaluated four Mendelian LDL-C genes—*LDLR, ABCG5, APOB*, and *PCSK*—for association with LDL and early-onset CHD risk in the population. About 1 person in 150 carries a deleterious mutation in *LDLR* or *ABCG5* mutation and the presence of such mutations is associated with a substantially higher LDL-C as well as a 3-fold increased risk for early-onset CHD. Remarkably, about 3% of all early-onset CHD is attributable to mutations in one of these two genes.

#### 3.2.2. Neuropsychiatric Genes

A growing number of genes and variants have been robustly associated with a range of neuropsychiatric disorders, and many of these genes have been shown to have pleiotropic effects across conventional diagnostic boundaries (e.g., [12]). *DRD2* has been identified as a genome wide significant locus for schizophrenia (SCZ), and DRD2 is the primary target of all approved antipsychotic drugs. The *DRD2* locus has also repeatedly been associated with risk of ADHD, addictions, and antipsychotic response. *CACNA1C* encodes a voltage-dependent L-type calcium channel subunit essential for normal cardiovascular and nervous system function. Rare missense mutations in exons 8 and 38 are a known autosomal dominant cause of Timothy Syndrome (OMIM 601005), which is characterized by long QT interval, syndactaly, developmental delay and autism. Significant association has been reported for common variation in *CACNA1C* with psychotic and mood disorders in both European and Asian ancestry populations, and with brain expression of the gene. In addition, the effects of psychostimulants (used in ADHD) and antidepressants appear to be partly mediated by *CACNA1C*. Two other high priority genes, *TCF4* and *CHD8,* are both potent regulators of gene expression in which rare missense and LoF mutations are associated with neurodevelopmental disorders, yet each has been associated with pleiotropic outcomes. Rare missense variants and structural variation that disrupt *TCF4* cause Pitt-Hopkins syndrome, which is characterized by facial dysmorphism, developmental delay, and autonomic dysfunction and are strong risk factors in ASD; whereas common variation of *TCF4* has been robustly associated with SCZ. *TCF4* has also been associated with Fuchs’ corneal endothelial dystrophy and primary sclerosing cholangitis, with recent data suggesting it is an important regulator of epithelial-mesenchymal transition. We initially identified haploinsufficiency of *CHD8* as a highly penetrant risk factor for ASD [13], and LoF mutations have been consistently identified in exome sequencing studies in autism spectrum disorder.

#### 3.2.3. Selection of Immune Disease Genes

GWAS in immune diseases have implicated several hundred genes as harboring susceptibility variants ranging in frequency from common to rare, and having a wide range of effect sizes, from fully penetrant to modest effects. These susceptibility genes fall within two categories: (1) those with a pleiotropic effect that may be involved in increasing the likelihood of autoimmune reactions in general and (2) those that direct an autoimmune reaction towards a specific target tissue. Here, we focus on variants with pleiotropic effects that are relevant to a large number of immune-related phenotypes including (1) *HLA DRB1:* a gene unparalleled in explaining a large proportion of the risk of immune diseases given the large effect sizes and high frequencies of these alleles [14]; (2) *IL23R*: implicated in multiple diseases and harboring rare and common susceptibility variants as a key, nodal receptor in a molecular pathway enriched in susceptibility genes for IBD, MS, and RA [15]; (3) *TYK2*: a gene harboring pleiotropic common variants [16] as well as rare variants causing primary immunodeficiency and risk of severe infections [17] because of altered kinase signaling that mediates the type I interferon response; (4) *TNFRSF1A*: with common and rare alleles that alter its coding sequence and associated with MS and periodic fever syndrome, this gene is a critical receptor for the TNFα a target of medications in IBD and RA that make MS worse and can trigger MS in a subset of treated patients [18] (5) *CTLA4*: has multiple pleiotropic common and rare variants with severe immune dysregulation resulting from a truncated receptor which lowers surface expression [19]. It has also been developed into a soluble receptor approved for RA treatment.

### 3.3. Implementation Research: Impact of Return of Genetic Results on Health Outcomes

To address the eMERGE III goals of returning clinically actionable genetic results to patients and evaluating the effect on health outcomes, the Partners eMERGE site proposed a randomized controlled trial of return of results (RoR) for a specific category of unanticipated genetic findings. Specifically, we will screen our entire Biobank population of 25,000 subjects for pathogenic variants in the genes *LDLR*, *APOB* and *PCSK9,* and conduct an exploratory trial in disclosing this information*.* Biobank participants with pathogenic variants in any of these genes will be offered enrollment into a randomized trial in which their finding will be CLIA confirmed, and in one arm, this result will be communicated to their physicians through the EMR, while in the alternative arm, results will not be CLIA confirmed or communicated for a year. Over the one year trial, we will collect outcomes through participant surveys and EMR queries to assess physician visits, laboratory testing, changes in medication prescriptions, LDL levels, medical costs and the number of family members screened and treated as a result of the intervention. We will collaborate with the entire eMERGE III network to incorporate what we learn from this pilot trial into large-scale implementation protocols for the genes selected by the network.

#### 3.3.1. Prior Experience with Return of Genetic Results at the Partners Site

Our investigative team has extensive experience with implementation of genetic RoR. For example, the Genomes2People (G2P) Research Program in Translational Genomics and Health Outcomes (PI: RCG) conducts clinical trials, and combines survey and epidemiological methodologies to generate novel scientific evidence around medical, behavioral and economic outcomes of using genetics and genomics. G2P research studies began 15 years ago with clinical trials that have examined the impact of disclosing single gene risk for common complex disease [20,21,22] and have continued to examine the impact of consumer genomics [23,24,25,26]. The G2P Program is now conducting randomized controlled trials assessing the impact of sequencing in the clinical care of adults (the MedSeq Project) [27,28,29] and of newborns (the BabySeq Project) (http://www.genomes2people.org/babyseqproject/). The G2P Research Program, along with others from PPM and from around the country, has contributed to current best practices in genomic medicine [30], and led a number of initiatives and policy statements around return of genomic results in both clinical and research venues [9,31,32,33,34].

PPM has also developed the GeneInsight Suite, an IT platform for the management and clinical communication of genetic results (see Aronson *et al.* [4]). Partners Healthcare has used the GeneInsight Suite to generate clinical genetic reports and deliver them to clinicians, organize results in rich EMR-linked displays, and provide clinical decision support. PPM has been delivering results to clinicians both inside and outside of Partners through GeneInsight Clinic since 2010 [35].

#### 3.3.2. Design of the Randomized Implementation Trial

**Figure 1:**
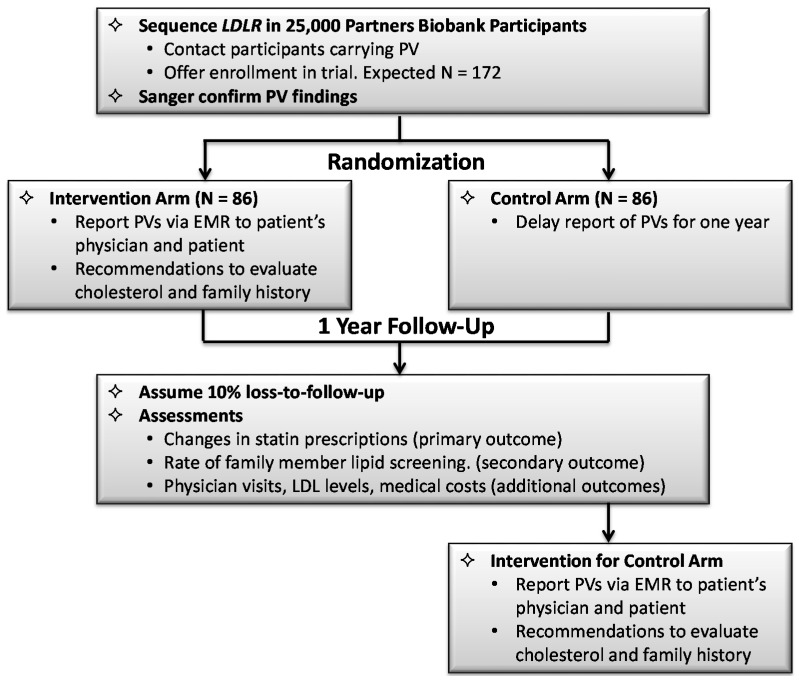
Design of implementation trial at the Partners eMERGE site.

For the purposes of the implementation trial, we chose to focus on rare variants in the *LDLR, APOB and PCSK9* genes, as these genes, particularly *LDLR*, underlie the most common causes of FH. Considerable data suggest that many patients with FH are undiagnosed and/or undertreated, resulting in many preventable MIs and deaths. While elevated lipids are readily detectable with laboratory testing, genomic screening of Biobank participants provides an opportunity to identify at risk patients in a situation where the EMR can be used to return these results immediately into clinical use for them, and alert their family that others may also be affected. Once a clinician is informed that his or her patient carries a PV suggestive of FH, family-based cascade screening for the same variants, or for elevated lipids can be recommended, potentially leading to the detection of unrecognized cases. (Figure 1).

Sequencing of the three FH genes will be performed for 25,000 DNA samples in the Partners HealthCare Biobank. Non-CLIA sequencing of these genes will be followed by bioinformatics filtering to identify LoF and missense variants most likely to be associated with high LDL-C. Novel missense mutations will be evaluated for pathogenicity using multiple computational algorithms. Biobank subjects ≥18 years old with PVs will be contacted and offered enrollment in the trial. If they agree, a separate blood sample for Sanger (CLIA) confirmation of the PV will be drawn, after which they will be randomized to (1) Intervention Arm: the PV in FH genes is communicated right away in the EMR through GeneInsight; or (2) Alternative Arm: communication of the PV is delayed for one year. Outcomes will be ascertained from RPDR and from the patients through quarterly surveys and interviews. The pre-specified primary outcome will be the number of patients in whom a relevant LDL-lowering medication, *i.e.*, statins, is started or the dosage is changed. The pre-specified secondary outcome measure will be the number of blood relatives that the patient reports have lipid levels tested and/or genetic testing as a result of the report to the patient’s physician. Additional outcomes will be LDL-C levels and number of physician visits. We will also evaluate cost-effectiveness of the intervention in terms of direct and indirect costs as well as measures of health related quality of life (e.g., QALYs).

## 4. Conclusions

The eMERGE III Network has brought together academic health centers around the nation to advance the integration of EMR-linked biorepositories and evaluate the impact of genetic information on health outcomes. The development of an eMERGE Clinical Center at Partners Personalized Medicine has been made possible through expertise and resources at the Partners site in EMR-based phenotyping, biobanking, and clinical implementation of genomic medicine. Genomewide common and rare variant genotyping of 25,000 participants in the Partners Biobank, targeted sequencing of clinically actionable genes through the eMERGE Network, and NLP-informed phenotyping in the longitudinal medical record will provide a powerful opportunity to characterize the penetrance and pleiotropy of risk variants across a spectrum of clinical conditions. In addition, a pilot randomized controlled trial of return of results for pathogenic variants in the three genes associated with FH will examine the impact of providing clinically actionable genetic data on healthcare utilization, cascade screening, health outcomes, quality of life indices, and empirically address ELSI issues. The discoveries that flow from this effort will inform the application of genomic information into EMRs for clinical research and best practices in clinical care using genomics.
